# Controlled spatial organization of bacterial growth reveals key role of cell filamentation preceding *Xylella fastidiosa* biofilm formation

**DOI:** 10.1038/s41522-021-00258-9

**Published:** 2021-12-07

**Authors:** Silambarasan Anbumani, Aldeliane M. da Silva, Isis G. B. Carvalho, Eduarda Regina Fischer, Mariana de Souza e Silva, Antonio Augusto G. von Zuben, Hernandes F. Carvalho, Alessandra A. de Souza, Richard Janissen, Monica A. Cotta

**Affiliations:** 1 Institute of Physics “Gleb Wataghin”, University of Campinas, 13083-859 Campinas, São Paulo Brazil; 2 Citrus Center APTA “Sylvio Moreira” Agronomic Institute of Campinas, 13490-970 Cordeirópolis, São Paulo Brazil; 3 Department of Structural and Functional Biology, Institute of Biology, University of Campinas, 13083-862 Campinas, São Paulo Brazil; 4 Kavli Institute of Nanoscience, Delft University of Technology, 2629 HZ Delft, The Netherlands

**Keywords:** Pathogens, Biofilms, Cellular microbiology

## Abstract

The morphological plasticity of bacteria to form filamentous cells commonly represents an adaptive strategy induced by stresses. In contrast, for diverse human and plant pathogens, filamentous cells have been recently observed during biofilm formation, but their functions and triggering mechanisms remain unclear. To experimentally identify the underlying function and hypothesized cell communication triggers of such cell morphogenesis, spatially controlled cell patterning is pivotal. Here, we demonstrate highly selective cell adhesion of the biofilm-forming phytopathogen *Xylella fastidiosa* to gold-patterned SiO_2_ substrates with well-defined geometries and dimensions. The consequent control of both cell density and distances between cell clusters demonstrated that filamentous cell formation depends on cell cluster density, and their ability to interconnect neighboring cell clusters is distance-dependent. This process allows the creation of large interconnected cell clusters that form the structural framework for macroscale biofilms. The addition of diffusible signaling molecules from supernatant extracts provides evidence that cell filamentation is induced by quorum sensing. These findings and our innovative platform could facilitate therapeutic developments targeting biofilm formation mechanisms of *X. fastidiosa* and other pathogens.

## Introduction

Bacteria have evolved diverse surface adhesion mechanisms to enable biofilm formation on biotic and abiotic substrates in a variety of natural, medical, and industrial settings^1–4^. The virulence of pathogenic bacteria strongly depends on their capability to attach to biotic surfaces and form multicellular assemblies^5–9^. Such pathogenic biofilms are highly resistant to diverse antimicrobial compounds due to their encapsulation within a matrix of hydrated extracellular polymeric substances (EPS)^8,10,11^. A better understanding of the mechanisms of bacterial adhesion and biofilm formation is thus vital to reveal potential vulnerabilities that can lead to their prevention and disruption^2,12–16^. In our previous work we characterized all individual stages in the process of biofilm formation in *Xylella fastidiosa*^17^, a vascular phytopathogen that causes large economical damage worldwide by inducing diseases in a range of important crops (e.g. citrus, grape, coffee, almond, olives, among others)^18,19^ and further shares genetic traits with human biofilm-forming pathogens^20,21^. With respect to the vital question of how large-sized biofilms are formed by this pathogen, we previously observed that cells during biofilm growth elongated up to 10-fold their typical size when connected with neighboring bacterial clusters, which we describe as a biofilm framework^17^. In this scenario, the extreme elongation of cells represents a central feature of biofilm formation, rather than simply a consequence of stresses such as starvation and DNA damage as commonly observed in other bacteria^22–26^. While similar filamentous cells have also been observed in *Vibrio cholerae*^7,14^, *Caulobacter crescent*^24^ and *Pseudomonas aeruginosa*^27^ during biofilm formation, solid evidence that such elongated cells are necessary for triggering and for the progression of biofilm formation remains fragmentary.

Whether filamentous cell growth of *X. fastidiosa* is the trigger rather than a consequence of biofilm formation remains a central question. Earlier results revealed that only a small fraction of cells undergo morphogenesis to filamentous cells^17^, which emanate from bacterial clusters. In this scenario, a stress-based trigger for filamentation does not seem to be present since such a stress would likely be shared by most cells in the cluster, and would therefore promote the morphogenesis of a majority of cells^22,23^. Another potential mechanism of triggering morphogenesis poses cell–cell communication via diffusible signaling factors (DSF) as part of the quorum sensing system. In particular, *X. fastidiosa* regulates its cell–cell adhesion, biofilm formation, and virulence in a cell density-dependent manner via DSF-based signaling^28–31^. A prime example of such quorum sensing regulation, the expression of adhesins as well as the secretion of outer membrane vesicles (OMVs), which contain biologically active biomolecules associated with cell functions linked to cell adhesion and virulence, occurs in a DSF-dependent, apparently cell density-dependent fashion^32–34^. Both adhesin expression and OMVs secretion in *X. fastidiosa* modulate its systematic dissemination in the host^34,35^. Therefore, since filamentous cells have been associated with biofilm formation, which itself is regulated by cell–cell communication^17^, we hypothesize that their formation is also a DSF-dependent process.

Since signaling molecules can strongly affect bacterial physiology and virulence, several methods for their in vitro and in vivo detection have been previously developed^36,37^. The majority of these methods rely on chromatographic and mass spectrometric techniques, or biosensor systems using genetically modified reporter bacteria^36^. However, such approaches include important limitations, particularly for signaling molecules used by *X. fastidiosa*. The detection of DSF molecules secreted by a small number of cells, such as when clusters are comprised of only a few cells, is difficult and requires methods capable of reliably detecting very low DSF concentrations. As such, assessing spatial gradients associated with filamentous cell formation poses an extreme challenge.

In this work, we circumvented these bottlenecks and elucidated the triggering mechanisms for *X. fastidiosa* cell filamentation during biofilm formation. Since the local DSF concentration produced by bacterial clusters decreases gradually and isotropically with distance, controlled spatial separation of bacterial adhesion is critical to ascertain its role in any process involving quorum sensing. Our previous works have suggested that *X. fastidiosa* has a higher adhesion affinity to gold than to other abiotic and biotic chemical substrates^5,17,38^. Here, we confirmed the high adhesion affinity of *X. fastidiosa* to gold and demonstrated that its spatial organization can be controlled by using lithographically defined gold patterns, therefore enabling the modulation of the size and distance between bacterial clusters. Exploiting its strong adhesion on gold, we were able to probe the formation of filamentous cells over an 18-h period and determine the effect of cell aggregate sizes and distance between spatially separated bacterial clusters. We demonstrated that the formation of filamentous cells is induced by local bacterial density; moreover, they are able to connect neighboring cell clusters in a distance-dependent manner, which eventually creates a network of interconnected cell clusters. The addition of supernatant extracted from highly dense cell cultures, which is abundant with diverse DSF^39,40^, demonstrated a significant increase in filamentous cell formation and cluster interconnections. Our results provide evidence that filamentous cell formation depends on a quorum sensing process. Likewise, the resulting formation of large-size biofilm frameworks composed of multiple interconnected cell clusters is also governed by quorum sensing, which may further represent an alternative mechanistic target for antimicrobials inhibiting biofilm-forming pathogens.

## Results

### *X. fastidiosa* preferentially adheres to gold surfaces patterns on silicon dioxide (glass)

**Fig. 1:**
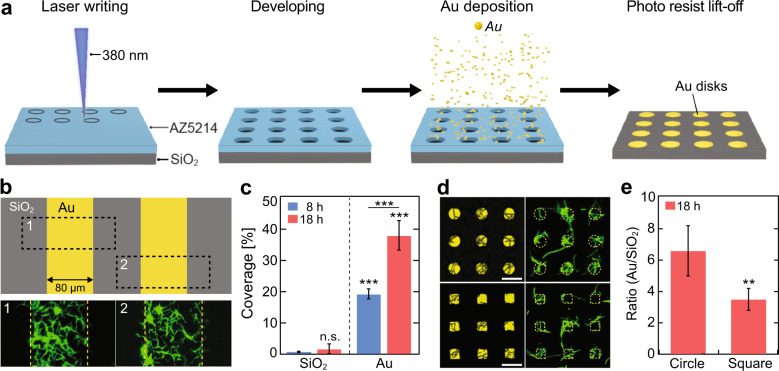
Fabrication of Au micropatterns and substrate-selective bacterial adhesion. **a** Stepwise schematic representation of Au pattern fabrication on SiO_2_ substrate. **b** Representative fluorescence images of selective bacterial adhesion to Au line patterns and SiO_2_ substrate after 18 h growth. **c** Bacteria coverage (mean ± s.d.) on SiO_2_ and Au line patterns after 8 and 18 h of growth. **d** Representative fluorescence and corresponding laser reflection images of bacterial adhesion on circular-shaped (11 µm diameter) and square-shaped (11 µm edge length) Au arrays, separated by 14 µm, after 18 h growth; scale bar denotes 20 µm. **e** Ratio (mean ± s.d.) of Au to SiO_2_ bacterial coverage on circular and square-shaped Au arrays after 18 h growth. Asterisks indicate statistical significance (**p < 0.01; ***p < 0.001; n.s. = non-significant) resulting from two-tailed, unpaired *t*-tests. See also Supplementary Fig. S1.

Since our previous work^5,17^ indicated that *X. fastidiosa* adheres more efficiently to gold than to diverse other biotic and abiotic surfaces, we first compared the propensity *of X. fastidiosa* to adhere to Au rather than to SiO_2_ as a function of time using quantitative assays. We fabricated Au micropatterns on SiO_2_ surfaces with different shapes and dimensions using direct-write laser (DWL) photolithography, followed by deposition of a 20 nm-thick Au coating using e-beam evaporation (Fig. 1a). After photo resist lift-off and cleaning, the substrates were sterilized with oxygen plasma prior to bacterial growth experiments.

On large Au areas with dimensions of 80 µm × 12 mm (Fig. 1b, top), we incubated GFP-expressing *X. fastidiosa* strain 11399^17,41^ for 8 and 18 h, after which non-attached bacteria were removed by gentle rinsing. Strikingly, widefield fluorescence microscopy (WFM) images revealed (Fig. 1b, bottom) that cells predominantly adhered to Au surfaces, even after 18 h of growth. The quantification of the bacterial coverage of equal areas of SiO_2_ and Au (Fig. 1c) revealed that significantly more (10–20-fold) cells adhered to Au as compared to SiO_2_, independent of growth duration. While cell adhesion to SiO_2_ was comparably low (<2% surface coverage) at both growth durations, a 2-fold higher bacterial coverage was observed after 18 h growth on Au compared to 8 h. These results not only support the previous finding that *X. fastidiosa* predominantly adhere to Au but also provide the means to create spatial patterns of bacterial colonies with controlled spatial separation.

With respect to potential variations in cell adhesion affinity to different surface pattern geometries, previous studies reported that rounded, circular shapes provide higher cell adhesion affinities for *Pseudomonas putida*, *Staphylococcus aureus,* and *Escherichia coli*^42,43^. Since a high cell adhesion affinity to the Au pattern is an important parameter for the intended goal of using this platform, we assessed whether particular Au pattern geometries also have an influence on the *X. fastidiosa* cell adhesion efficacy. To do so, cell adhesion affinity was tested with circular-shaped and square-shaped Au micropatterns (Fig. 1d, left), which were incubated with *X. fastidiosa* for an extended time of 18 h. Importantly, the formation of elongated, filamentous cells was readily observable on both Au pattern geometries (Fig. 1d). In order to quantitatively determine the cell-adhesion propensity on circular and squared Au shapes, either confocal laser scanning microscopy (CLSM) reflective images or WFM bright field images, together with their corresponding fluorescence microscopy images, were processed into binary images (Supplementary Fig. S1a–d) and subtracted from each other to determine the cell coverage on Au and SiO_2_ separately. We then calculated the cell coverage ratios of Au to SiO_2_ (Fig. 1e) from the cell coverages measured for each shape pattern (Supplementary Fig. S1e). The results (Fig. 1e) clearly show a 2-fold higher cell coverage ratio for circular-shaped than for square-shaped Au arrays. Circular-shaped Au arrays were thus used to probe distance-dependent and density-dependent formation of filamentous cells.

### Optimizing Au disk diameter, distance, and bacteria growth time to probe filamentous cell formation process

**Fig. 2:**
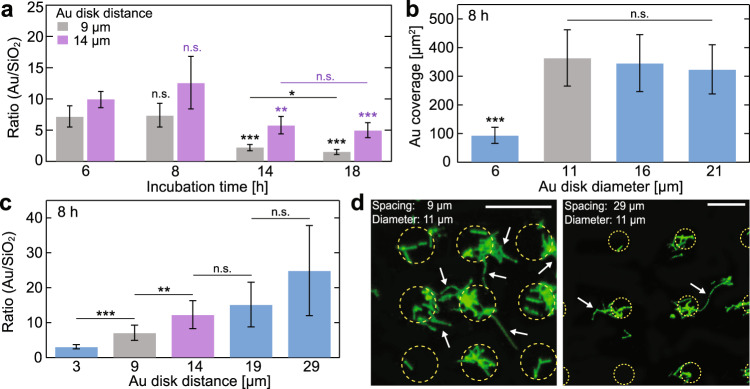
Substrate-selectivity of bacterial adhesion in dependency of growth time, Au disk diameter, and Au pattern distance. **a** Bacterial adhesion ratio (mean ± s.d.) of Au to SiO_2_ of circular Au disks arrays with 11 µm diameter, separated by 9 and 14 µm spacing for different growth times (6, 8, 14, 18 h). **b** Bacterial coverage (mean ± s.d.) on different Au disk diameters (6, 11, 16, 21 µm) with 9 µm spacing for 8 h growth time. **c** Bacterial adhesion ratio (mean ± s.d.) of Au to SiO_2_ of circular Au disks arrays with 11 µm diameter, separated by different spacing (3, 9, 14, 19, 29 µm) for 8 h growth time. **d** Representative fluorescence images of *X. fastidiosa* cells adhering to circular Au disks arrays with 11 µm diameter, separated by 9 µm (left) and 29 µm (right) spacing for 8 h growth time; scale depicts 20 µm. Asterisks indicate statistical significance (*p < 0.05; **p < 0.01; ***p < 0.001; n.s. = non-significant), resulting from two-tailed, unpaired *t*-tests. See also Supplementary Fig. S2.

The effect of growth duration, Au disk diameter, and distance on both the cell adhesion efficiency and substrate-dependent cell adhesion specificity was assessed. We probed samples grown for longer than that of the typical *X. fastidiosa* division time^17^ of ~6 h, namely 6, 8, 14, and 18 h. Using an Au disk diameter of 11 µm, as used in our substrate-selectivity experiments (Fig. 1d, e), we simultaneously examined the effect of different growth durations and Au pattern distances. Here, we used 9 and 14 µm separation distances, representing values larger than approx. 2-fold and 3-fold the typical length of *X. fastidiosa* cells (~3–4 µm)^18^, respectively. We reasoned by this approach to be able to readily discriminate between normal cell lengths and those of filamentous cells. Remarkably, independent of growth durations and Au pattern distances, the propensity of cells to adhere to Au rather than SiO_2_ remained (Fig. 2a).

However, when the typical *X. fastidiosa* division time of ~6 h was largely exceeded (14 and 18 h incubation), a considerable number of ≥3rd generation daughter cells were encountered on the SiO_2_ substrate, and the substrate selectivity seemed reduced. Since the cell adhesion ratios of Au relative to SiO_2_ between 6 and 8 h, and between 14 and 18 h, were statistically comparable for both Au pattern distances, subsequent experiments were carried out using 6–8 and 18 h growth durations. We reasoned that the 6–8 h growth duration may resemble the initial stages of biofilm formation dictated primarily by adhesion of planktonic cells, whereas 18 h reflected the detection of slow-growing filamentous cells as the cell mass adhering to SiO_2_ (e.g. Fig. 1d).

Yet another possibility causing *X. fastidiosa* cells adhering to SiO_2_ could result from a limiting Au area for further planktonic and daughter cell adhesion, resulting in increased adherence to SiO_2_. To verify this possibility, we varied the Au disk diameter in our arrays, ranging from 6 to 21 µm in increasing steps of 5 µm (which corresponds to the upper limit for typical cell lengths^18^). Indeed, while Au disks with diameters ≥11 µm exhibited similar cell coverage (~370 µm^2^), smaller disks with a diameter of 6 µm (Fig. 2b) showed a 4-fold lower cell coverage (~90 µm^2^). Although expected from the different disk areas, this result also indicated that small Au disk areas can limit bacterial coverage; we proceeded with 11 µm disk diameters for all subsequent experiments, since this limiting effect was not observable for Au disk diameters ≥11 µm.

If indeed potential cell–cell communication via quorum sensing mediates the formation of filamentous cells^8,44,45^, we reasoned that variations in distance between Au disks should affect this process. Following this idea, the formation of filamentous cells was monitored via fluorescence imaging of cells clusters separated over various distances. Therefore, *X. fastidiosa* was incubated on Au disk patterns with distances ranging from 3 to 29 µm (Fig. 2c). As observed before, the cell adhesion propensity was higher on Au than on the SiO_2_ substrate, but, in turn, the cell coverage ratio of Au to SiO_2_ also increased with separation distance. This result indicates that the cell mass adhering to SiO_2_ between the Au disks, which predominantly consists of both elongated and filamentous cells (Fig. 2d), decreased with distance. A distance of 3 µm between Au disks was unusable in the study of the formation of filamentous cells since the typical cell size of 3–4 µm^18^ already bridged the SiO_2_ area between neighboring Au disks upon cell adherence (Supplementary Fig. S2). More strikingly, significant differences in the Au to SiO_2_ ratios were encountered between 9 and 14 µm disk separation, but not in the case of larger distances (19 and 29 µm). This observation rendered Au disk distances of 9 and 14 µm ideal for systematic investigation of bacterial cluster proximity and size in the formation of filamentous cells.

### The formation of filamentous cells depends on bacterial cluster density and distance

**Fig. 3:**
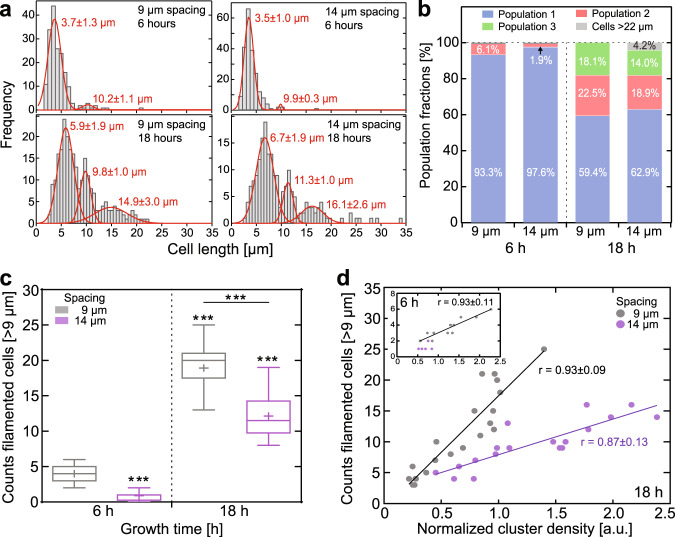
Bacterial cluster distance-dependent and density-dependent formation of filamentous cells. **a** Cell length distributions for 9 and 14 µm spacing, grown over 6 and 18 h (6 h: 9 µm, *N* = 183; 14 µm, *N* = 213; 18 h: 9 µm, *N* = 249; 14 µm, *N* = 191). Gaussian fits (red) resulting from the applied Gaussian mixture exhibit three distinct cell length populations (see also Supplementary Fig. S3a); the fit values (±s.d.) are denoted within the plot. **b** Fractions (integral of Gaussian fits from (**a**)) of the different populations observed in all tested conditions. **c** Number of filamentous cells detected in the proximity of-or emanating from-bacterial clusters for all tested conditions (from *N* = 9 fluorescence images each). Legend for box and whisker plot: the center line denotes the median value (50th percentile), the cross the mean average, the bounds of the box contain the 25th to 75th percentiles, the whiskers mark the 1.5 interquartile range. **d** Number of filamentous cells in dependency of cluster density after 18 h growth (inset for 6 h growth), calculated from the bacterial cluster fluorescence intensity and normalized to the number of filamentous cells for all tested conditions. Lines represent linear fits and the corresponding Pearson correlation coefficients (*r*) are denoted for each fit. Asterisks indicate statistical significance (***p < 0.001), resulting from two-tailed, unpaired *t*-tests. See also Supplementary Fig. S3.

To evaluate whether the density of cell aggregates or their proximity is the decisive parameter for filamentous cell formation, we analyzed all *X. fastidiosa* cell lengths in samples with 9 and 14 µm Au disk distances and grown over 6 and 18 h. Notably, the cell length distribution over all samples (Fig. S3a) revealed the existence of three distinct cell length populations. The cell length distributions for each of the four tested conditions (Fig. 3a) exhibited a similar picture with three populations of comparable average length values. After 6 h of cell growth (Fig. 3a, top graphs), where predominantly planktonic cells adhere and at most one cell division occurs, the dominant population exhibited an average cell length of ca. 3–4 µm, independent of the Au disk distance (Fig. 3b, left).

While this average cell length is comparable to that of a typical *X. fastidiosa* cell^12^, a minor secondary population of filamentous cells was found with an average cell length of ~10 µm (Fig. S3c, center). The increase in cell length was more distinct after 18 h cell growth (Fig. 3a, bottom): while the subpopulation with average cell lengths ~10 µm strongly increased in proportion, a third population of filamentous cells with an average cell length of ~15–16 µm appears (Fig. 3b). This third subpopulation exhibited a 4-fold increase in cell length compared to a typical *X. fastidiosa* cell of 3-4 µm (Fig. S3c, right).

Interestingly, the average cell length after 18 h growth exhibited strong distance-dependent increase, with significantly larger cells observed for samples separated by 14 µm (Fig. S3c). Under this condition, an additional small subpopulation of very long filamentous cells with lengths >22 µm emerged (Fig. 3a, bottom right; Fig. 3b). Given that 14 µm pattern distance results in a diagonal distance between the Au disks of ~20 µm, the appearance of filamentous cells of such lengths were to be expected. Both the increase in filamentous cell length and the appearance of very long cells >20 µm in case of 14 µm pattern distance might imply that the growth of filamentous cells, predominantly emanating from cluster boundaries, might be directed towards and connect adjacent clusters, as we indeed observed in fluorescence images (Supplementary Fig. S3b).

We next sought out to determine whether the formation of filamentous cells depends on growth duration and spatial distance between cell clusters. For this analysis, it was necessary to define a cell length that allows discriminating cells as being filamentous or not. Since the dominant cell length population after only 6 h of cell growth predominantly represents non-elongated cells with sizes typically encountered for *X. fastidiosa*^18^, the second population, which increases in relative abundance after extended growth duration, was deemed to consist largely of filamentous cells. We thus defined cells with lengths of 9 µm or larger, corresponding to the one-sigma lower bound of the second population distribution (Supplementary Fig. S3a), as filamentous cells. Upon quantifying the number of filamentous cells in all tested conditions, we found up to a 5-fold higher abundance of filamentous cells after 18 h growth than we observed after 6 h growth (Fig. 3c). Importantly, while longer growth duration increased the occurrence of filamentous cells independent from spatial separation, the number of filamentous cells notably decreased with the spatial distance between cell clusters.

Since extended growth duration led to an increase of filamentous cell formation, we questioned whether the cell density that increases with time, rather than growth duration itself, is the decisive parameter. To address this question, we estimated the bacterial cluster densities by integrating their fluorescence intensity by taking into account that integrated fluorescence intensity scales linearly with cell density. This method was also deemed appropriate since the long *X. fastidiosa* cell-division time of ~6 h^17^ allows the observation of the horizontal expansion of cells within our observation times before the formation of large 3D biofilm architectures. Interestingly, our results revealed (Fig. 3d) a strong correlation (Pearson correlation coefficient *r* ~ 0.9) between the abundance of filamentous cells and the abundance of cells within clusters. However, and more importantly, while there was no effect of growth duration on filamentous cell formation observable, we detected that the distance between clusters had a significant effect (Fig. 3d) on filamentous cell formation. Larger distances between clusters (14 vs. 9 µm) resulted in significantly less filamentous cells, even at high cell densities. These results confirm our hypothesis that filamentous cell formation depends on both cell density and their spatial separation.

### Filamentous cells interconnect bacterial clusters to form a biofilm framework

**Fig. 4:**
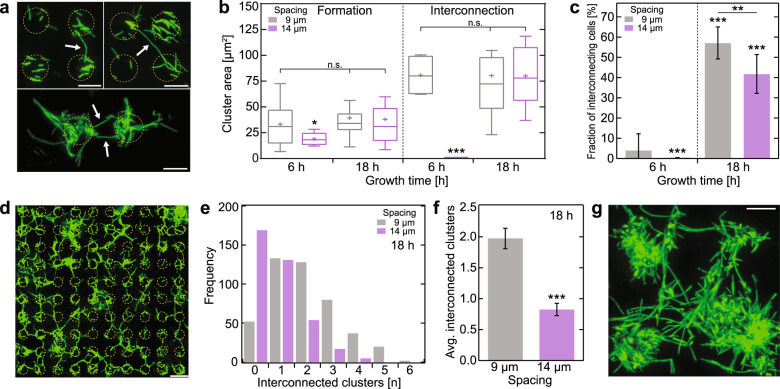
Filamentous cell growth is directed to adjacent bacterial clusters and interconnect cell clusters to a large network. **a** Example fluorescence images of bacterial cluster pairs interconnected by filamentous cells, indicated by arrows; scale bar depicts 10 µm. **b** Bacterial cluster area-dependent *formation* of filamentous cells and filamentous cells that *interconnect* adjacent clusters for 9 and 14 µm spacing, grown for 6 and 18 h. Cluster size is normalized to the number of filamentous cells. Legend for box and whisker plot: the centerline denotes the median value (50th percentile), the cross the mean average, the bounds of the box contain the 25th-75th percentiles, the whiskers mark the 1.5 interquartile range. **c** Fractions (mean ± s.d.) of *interconnecting* cells of all filamentous cells observed for 9 and 14 µm distances, grown for 6 and 18 h. **d** Example fluorescence image of an Au disk array with 9 µm spacing after 18 h growth shows multiple interconnected bacterial clusters; scale bar depicts 20 µm. **e** Degree of interconnected clusters (*N* = 446 for 9 µm, *N* = 372 for 14 µm distances) and **f** average number (mean ± 95% confidence interval) of cluster interconnections of 9 and 14 µm distances arrays after 18 h growth. **g** In-vitro CLSM fluorescence image of growing biofilm, originating from clusters interconnected with multiple filamentous cells; scale bar depicts 20 µm. Asterisks indicate statistical significance (*p < 0.05; **p < 0.01; ***p < 0.001; n.s. = non-significant), resulting from two-tailed, unpaired *t*-tests. See also Supplementary Fig. S4.

Our previous work had raised the concept that the underlying function of filamentous cells is to connect spatially separated cell aggregates to form the macroscale network that is required for the formation of subsequent large-scale mature biofilms^17^. Our observation of filamentous cells that interconnect adjacent bacterial clusters (Fig. 4a) supports this model. We reasoned that the growth of filamentous cells might be directed towards adjacent bacterial clusters and is potentially governed by a quorum-sensing mechanism, triggered by locally high concentrations of DSF in the surrounding environment of clusters. If this model is true, we would expect that both cell cluster size and the distance between clusters would drive filamentous cells to connect adjacent cell aggregates. To verify this model, we differentiated between *formed* filamentous cells (originating from single-cell clusters) and *interconnecting* (bridging two or more cell clusters) filamentous cells. Since we found that filamentous cell formation depends on cell cluster size and separation distance, we determined the occurrence of the two classes of filamentous cells as a function of these two parameters.

In agreement with our prior observation, the *formation* of filamentous cells predominantly depends on a minimal bacterial cluster size, independent of growth time or cluster distance (Supplementary Fig. S4a), with ~35 µm^2^ coverage in average. More strikingly, the formation of *interconnecting* filamentous cells also exhibits a strong cluster-size dependency, since we only observed those in much larger cell clusters (≥80 µm^2^). Upon discriminating between growth duration and spatial distances between cell clusters (Fig. 4b), we observed that no *interconnecting* filamentous cells were formed after 6 h growth when separated by 14 µm, but otherwise the cluster sizes leading to either *forming* or *interconnecting* filamentous cells are comparable. Expectedly, the fraction of *interconnecting* cells increased significantly with growth duration (Fig. 4c), mainly due to the associated increase in cell cluster size; after 18 h growth, ~50% of all filamentous cells were *interconnecting* cells connecting adjacent clusters. However, the fraction of *interconnecting* cells after 18 h growth was significantly lower (~16%) for clusters 14 µm apart compared to those separated by 9 µm. The dependency of cluster-*interconnecting* filamentous cells on cell cluster size and spatial distance indicates that a diffusion-dependent process, such as DSF-dependent quorum sensing, may trigger filamentous cell formation.

Particularly after 18 h growth, we observed many cell clusters interconnected with each other, suggesting that filamentous cells connect multiple adjacent clusters to form a large-scale network (Fig. 4d). Upon analyzing the interconnections between all cell clusters (Supplementary Fig. S4b), we were able to quantify the number of adjacent cluster interconnections per cell cluster (Fig. 4e) for the two tested Au pattern distances (9 and 14 µm). The frequency distributions of the observed number of interconnections per cluster (Fig. 4e) exhibited in the case of 14 µm Au disk distances up to six interconnected cell clusters, whereas in the case of 9 µm Au disk distances, the maximum observed number of interconnected clusters was 4. The difference in the degree of cluster interconnections as a function of cluster distances is more apparent upon comparing the average number of interconnected clusters (Fig. 4f). Here, the average number of interconnections per cell cluster at the 14 µm cluster distance was ~2-fold lower (~0.8) than that observed for cell clusters spaced 9 µm apart (~1.9). This striking result demonstrates that the degree of cell cluster interconnections is inverse to the spatial cluster distance, as we initially hypothesized. This result, together with the observation that multiple interconnections between large clusters were readily observable in biofilms (Fig. 4g), support the idea that the formation of filamentous cells ultimately lead to large cluster networks that assemble into large-scale biofilms during its maturation.

### Culture supernatant extract increases filamentous cell formation and cluster interconnection

Triggers of phenotypic changes can originate from starvation^23,24^. To probe the possibility that nutrient deprivation may induce filamentous cell growth, we repeated the 8 and 18 h cell growth experiments under the optimal conditions found for investigating cell cluster density- and distance-depending filamentous cell formation (Au disk diameter: 11 µm, Au disk pattern distances: 9 and 14 µm) with nutrient replenishment. After half of the culture growth time (4 and 9 h for total growth times of 8 and 18 h, respectively), 20% (v/v) of fresh PW growth media was added to the samples. The results show that the replenishment of PW growth media did not affect the overall substrate-specific cell adhesion propensity nor the cell cluster density- and distance-dependency in inducing filamentous cell growth (Fig. S5a, b and S5c, d, respectively). This observation confirmed that filamentous cell growth was not caused by mechanisms of starvation.

We next sought out to verify the prior indications of diffusion-dependent quorum sensing being a potential factor involved in triggering filamentous cell formation. To overcome the practical limitations for this particular pauca strain in creating viable knock-out variants (relevant here for the DSF synthase Δ*rpfF* and sensor kinase Δ*rpfC* genes)^29,30^, as well as the current deficiency in knowledge about the exact number and function of *X. fastidiosa*-specific DSF molecules^32^, we turned to adding cell culture supernatant extracts to our growth media. We reasoned that a supernatant extracted from a 10-day-old growth of highly dense *X. fastidiosa* cell culture should be enriched with a high concentration of diverse quorum sensing-associated DSF molecules, based on a protocol that has been successfully applied to *X. fastidiosa* and other biofilm-forming bacterial pathogens in the investigation of quorum sensing-associated mechanisms^29,39,40,46,47^.

We kept the same optimized experimental approach and Au pattern parameters as used for the experiments without supernatant and for the nutrition deprivation experiments described above but added 2% (v/v) of supernatant extract solution after 4 and 9 h of growth (for the 8 and 18 h total growth time, respectively). We note here that higher concentrations of the supernatant extract resulted in extremely large and dense cell clusters after a few hours, rendering reliable investigation of filamentous cell formation unfeasible. Related to this observation, the overall cell adhesion propensity on both Au and SiO_2_ significantly increased (Fig. S6a) upon adding 2% (v/v) supernatant solution, while the substrate selectivity for Au remained unchanged (Fig. S6b). Such increased overall cell adhesion propensity is in agreement with previously reported observation for the *X. fastidiosa* subsp. *fastidiosa* Temecula strain, where Δ*rpfC* mutants overexpressed DSF molecules^48^. The associated increase in cell cluster area as a function of the *formation* and *interconnecting* filamentous cells was also observable (Fig. S6c), particularly after 18 h growth time with supernatant acting on cells for 9 h, and was independent from the cell cluster distance.

**Fig. 5:**
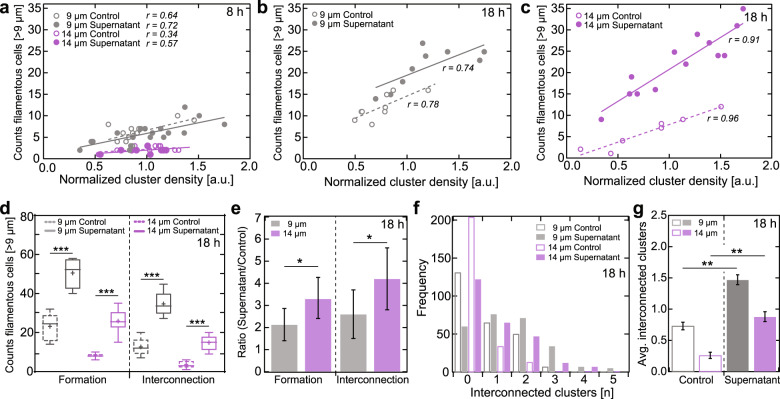
Supernatant extract decreases cluster distance- and density-dependency in the formation of filamentous cells. **a** Number of filamentous cells in dependency of cluster density after 8 h growth in the absence (control) and addition of 2% (v/v) supernatant (SN) after 4 h for 9 and 14 µm distances. **b**, **c** Same as (**a**), but with a growth time of 18 h and the addition of SN after 9 h for **b** 9 µm and **c** 14 µm distances; analyzed as in Fig. 3d. **d** Number of filamentous cells (Formation) and cluster-interconnecting cells (Interconnection) for 9 and 14 µm distances (*N* = 5 images each), grown for 18 h in absence (control) and addition of 2% (v/v) of SN after 9 h. Legend for box and whisker plot: the centerline denotes the median value (50th percentile), the cross the mean average, the bounds of box contain the 25th to 75th percentiles, the whiskers mark the 1.5 interquartile range. **e** Ratio (mean ± s.d.) of the number of filamentous cells (Formation) and cluster-interconnecting cells (Interconnection) in the absence (control) and addition of 2% (v/v) of SN after 9 h for 9 and 14 µm distances, extracted from (**d**). **f** Degree of interconnected clusters (*N* = 256 for each shown data set) and **g** average number (mean ± 95% confidence interval) of cluster interconnections of 9 and 14 µm distances after 18 h growth in absence (control) and addition of 2% (v/v) SN after 9 h. Asterisks indicate statistical significance (*p < 0.05; **p < 0.01; ***p < 0.001; n.s. = non-significant), resulting from two-tailed, unpaired *t*-tests.

The prior identified traceable parameters, cell cluster density and distance, associated with filamentous cell formation, did not show any noticeable change for a supernatant residence time of 4 h during the total of 8 h cell growth (Fig. 5a) in case of both 9 and 14 µm cluster distances. Conversely, with a ~2× longer residence time of the added supernatant of 9 h during a total of 18 h growth, the already high number of filamentous cells formed on substrates with 9 µm cluster distance increased twofold over the entire cell density range (Fig. 5b). The effect of 9 h supernatant residence time on substrates with cluster distances of 14 µm was even more striking: while in all prior experiments the number of formed filamentous cells was consistent ~2× lower than for 9 µm cluster distances over the entire cell density range, the addition of supernatant increased the formation of filamentous cells four-fold on a global level (Fig. 5c), even exceeding the number of filamentous cells detectable for 9 µm cluster distances with respect to cell density. The vast stimulation of filamentous cell *formation* by the added supernatant was also observable in the similarly significant increase of *interconnecting* cells on the global scale in the case of 9 and 14 µm cluster distances (Fig. 5d, e). The resulting degree of interconnected cell clusters, which is vital for laying the basic framework for biofilm maturation, exhibited a significant increase in the number of cell cluster interconnections (Fig. 5f, g); for 9 µm cluster distance, the number of interconnected cells cluster doubles, while for 14 µm distances the increase was three-fold.

The results support our hypothesis of a DSF-dependent trigger of filamentous cells formation, regulated by diffusion-dependent quorum sensing mechanisms: the artificial increase of the global DSF concentration would induce both an overall higher number of filamentous and interconnecting cells. It would also induce the formation of filamentous cells for isolated clusters where the surrounding DSF concentration is natively lower, which is particularly apparent for the 14 µm cluster distance.

## Discussion

Our study elucidates the phenomenon by which cell filamentation occurs during bacterial biofilm formation, and reveals that their formation is involved in the creation of interconnected large cell cluster networks that lay the foundation of macroscale biofilms. Our developed platform to systematically investigate potential triggers and function of filamentous cell morphogenesis demonstrated that local cell abundance is the predominant parameter inducing cell filamentation, consistent with the model in which a cell density-dependent gradient of diffusible signaling molecules drives morphogenesis. In the following, we discuss the most relevant findings and implications.

### High affinity for cell adhesion to gold enables noninvasive, controlled spatial cell patterning

The selective and controlled spatial arrangement of cells and microcolonies are critical prerequisites for the investigation of dynamic biological phenomena of multicellular systems, such as cell plasticity, motility, morphogenesis, and cell–cell communication^49–51^. To this end, diverse approaches for selective cell organization with varying complexity and different surface chemical modifications have been previously developed^12,42,51–53^. To simplify the approach, we exploited the native ability of *X. fastidiosa* to bind to specific chemical moieties with high affinity. Previous observations of the growth of *X. fastidiosa* on several different materials, such as Si, SiO_2_, InP, Au as well as various biotic surfaces mimicking the host environment, i.e., ethyl cellulose and cellulose acetate^5,38^, indicated a higher adhesion affinity to Au surfaces. Our results confirmed this phenomenon, demonstrating that planktonic cells, as well as daughter cells after multiple cell divisions, predominantly adhered to gold^54^. Our results further revealed a significant preference of cells to adhere to circular shapes over squared geometries, a characteristic that has also been previously observed for diverse bacteria and surface compositions^42,55^.

While bacterial cells predominantly adhered to gold deposits in our experiments, the number of cells adhering to the SiO_2_ interspace decreased with pattern distance. This rather non-intuitive observation might originate from the ability of *X. fastidiosa* to move along surfaces, at speeds up to 5 µm/min and against the flow, via type IV pili-mediated twitching-motility^56^. Due to the taxis ability of *X. fastidiosa*, cells outside the Au areas might either randomly move until reaching the Au patterns or even move directed to regions of higher cell amounts by chemotaxis (involving quorum sensing), which has been observed for other bacterial species^57–60^. The high affinity to gold is most likely mediated by its membrane protein methionine sulfoxide reductase that forms disulfide bonds to thiol groups on surfaces and adjacent cells^61,62^. In fact, membrane-associated thiol groups have been found in adhesion proteins and are key molecules involved in the adhesion mechanism of several bacterial species, including human pathogens^61,63,64^. Exploiting the strong interactions between gold and membrane-associated thiol groups^65^, coupled with the advantage that gold is biocompatible, chemically inert, and commonly used in biomedical applications^66,67^, our development provides a facile platform to create spatially well-defined cell adhesion patterns for noninvasive cell studies. Our methodology thus readily enables the systematic study of various complex phenomena involved in biofilm formation^48^ for a broad range of plant and animal pathogens^44,68,69^.

### Formation of cluster-interconnecting filamentous cells enables the creation of large biofilms

Our observed systematic dependency on cell cluster density and cluster distance in the formation of filamentous cells supports an earlier finding that first described the existence of filamentous cells and suggested that they might play a key role in the formation of large biofilm architectures by interconnecting cell clusters^17^.

One of the most intriguing results of our study revealed that filamentous cells can grow until reaching neighboring cell clusters, where they eventually integrate themselves into the reached cluster. This growth behavior is unprecedented in bacterial pathogens, albeit suggested as *X. fastidiosa* cell clusters have been previously observed to be interconnected by filamentous cells^17^. Our observation that the filamentous cell growth can form interconnections with proximal clusters depended strongly on the distance between the clusters, which in turn suggests that the extent of filamentous growth might be governed by an extracellular regulatory mechanism. The possible linkage to quorum sensing of this process is attractive since DSF gradients produced by cell clusters could explain the initiation of cell morphogenesis. Indeed, our results demonstrate that supernatant extracted from highly dense cell cultures, abundant with a large spectrum of DSF molecules^29,39,40^, induces the formation of filamentous cell phenotypes and increases, in turn, the degree of interconnected cell cluster network. This intriguing observation establishes that the processes of cell filamentation are regulated by a quorum-sensing mechanism.

**Fig. 6:**
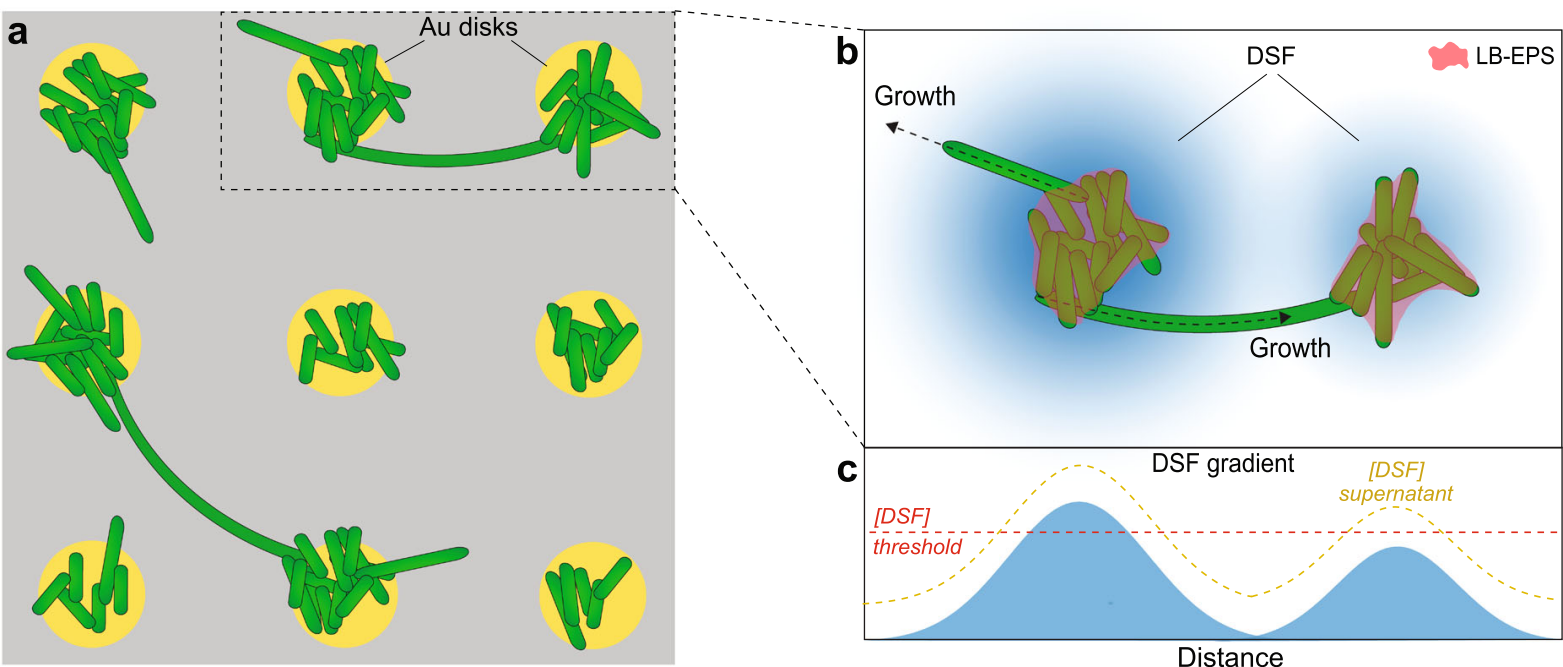
Proposed model of DSF-regulated filamentous cell formation and cluster interconnection. **a** Schematic representation of experimentally observed filamentous cells interconnecting adjacent cell clusters. **b**, **c** High, cluster density-dependent local DSF concentration (in blue) above a certain DSF concentration threshold (**c**, dashed red line) triggers filamentous cell growth of non- or partly-EPS covered cells localized at cluster boundaries. The addition of supernatant extract abundant with diverse DSF increases the global DSF concentration (**c**, dashed yellow line) resulting in filamentous cell formation also at clusters with prior insufficient DSF concentration (see also Fig. 5), such as those separated by 14 μm distance (Fig. 5c).

Despite the expectation that the DSF-mediated induction of cell filamentation would affect all cells in clusters of any size, our results exhibited that filamentous cell growth emerges preferentially from cells localized at cluster boundaries. In all of our experiments, only a small fraction of cells at cluster boundaries undergo cell morphogenesis and resembles largely the indications of our previous observation that similarly noted few filamentous cells in randomly nucleated bacterial clusters and small biofilms^17^. Taking into account that cell clusters become encapsulated in EPS during biofilm formation (i.e. loosely bound EPS)^17,70^, cells at cluster boundaries might either not be entirely covered with EPS or the EPS layer is still thin enough to not fully act as DSF diffusion barrier, rendering the cells susceptible to regulatory quorum sensing (Fig. 6). A previous study that demonstrated that EPS can act in quorum sensing signal retention supports this model^71^. However, further investigation is warranted to elucidate how EPS can act as a potential DSF diffusion barrier, and to what extent.

With respect to the underlying function of cluster-interconnecting filamentous cells, we observed that cells that had undergone such phenotypical morphogenesis eventually formed large networks of many interconnected bacterial clusters preceding the formation of larger biofilms. This finding confirms our original assumption that cluster-interconnecting filamentous cells form a network that can facilitate spatial biofilm extension and its maturation^17^. During this process, continuous secretion of soluble EPS (i.e. S-EPS)^17^ by such filamentous cells could further provide an organochemical conditioning film between the clusters that promotes cell adhesion^17^.

Analogous to our findings, the reinforcement of cell surface adhesion has been found associated with filamentous cell formation during biofilm development for the pathogens *V. cholerae*^7^, *P. aeruginosa*^27^, and *X. fastidiosa* Temecula^30^. The link between DSF signaling and surface adhesion in *X. fastidiosa* Temecula has been identified based on the observation that DSF overexpression by a Δ*rpfC* DSF sensory kinase mutant significantly increased the cell holdfast to the surface. Our observation that the cell surface adhesion significantly increased in presence of the supernatant extract indicates that DSF signaling is also involved in surface adhesion propensity and filamentous cell formation in our used pauca strain.

### Bacterial density-dependent cell communication regulates filamentous cell growth

Biofilm-forming bacteria commonly communicate by means of a cell density-dependent mechanism known as quorum sensing^72–74^. Quorum sensing has been identified to regulate bacterial adhesion, virulence, gene expression, resistance, and other traits, rendering this process vital for lifestyle adaptation in diverse pathogens^75–81^. In the case of *X. fastidiosa*, it has been similarly shown that cell–cell adhesion, virulence, and biofilm formation are regulated by the secretion of DSF molecules in a cell-density-dependent fashion according to insect and plant hosts^28–30,32,45^.

The concept of cell density-dependent changes in cell behavior and phenotypes by DSF-based quorum sensing underlies our observed cell density-dependent initiation of filamentous cell morphogenesis^17,45,82^. Supporting this notion was the fact that the global enrichment of DSF molecules in our experimental assay—originating from highly dense culture supernatant-induced vastly the filamentous phenotype.

In further support to this conjecture, previous observations in *X. fastidiosa* as well as in other bacteria and human cells have shown that quorum-sensing mechanisms are activated upon exceeding a local DSF concentration threshold at regions of high cell densities^39,51,74,83,84^ Consistent with this model, previous studies showed that a *X. fastidiosa* Δ*rpfF* DSF synthase mutant, with blocked production of DSF, was unable to form biofilms in an insect vector, whereas the Δ*rpfC* DSF sensory kinase mutant was unable to react to the DSF signal resulting in phenotypes unable to spread in the plant resulting in vastly reduced virulence^29,30^. Considering our experimental results, we reason that the inability to form biofilms in either of the two habitats might originate from the combination of a decreased capability of increasing the cell-surface holdfast and the formation of interconnecting clusters, both induced by DSF-mediated quorum sensing processes.

Combining our observations with previous findings of quorum-sensing processes in *X. fastidiosa* and other bacterial species, we propose a model of DSF concentration-dependent initiation of cell filamentation and cell cluster interconnection for *X. fastidiosa* (Fig. 6). Once a cell cluster is sufficiently dense to produce a local DSF concentration that passes a certain threshold, cells at the outer cluster boundaries, which are not or not fully encapsulated by EPS (i.e. LB-EPS^17^), may undergo cell morphogenesis to filamentous cells. Our observation that a notable number of filamentous cells stopped elongating before reaching adjacent cells clusters implies that initial cell growth occurs in random directions. Moreover, the fact that cluster-interconnecting cells are often oriented towards nearby clusters might indicate that filamentous cells can somehow sense and grow towards a higher DSF gradient. Such dependency on spatial proximity between bacterial cell clusters in quorum-sensing efficacy has also been found earlier in other bacterial species^39,74,84^. However, our experimental results so far are inconclusive in this matter and warrant further investigation with a more specifically designed platform and experimental assay.

The question of which of the three currently known DSF molecules (XfDSF1, XfDSF2, CVC-DSF)^32^ might be responsible for triggering cell morphogenesis is currently difficult to answer since filamentous cell growth is a rather new observation and the exact function of the DSF molecules within the *X. fastidiosa* lifecycle have not yet been fully identified. The potential creation of a DSF expression knock-out mutant to probe the function of these DSFs is particularly difficult in the case of the used pauca strain as the cell fitness and stress responses are highly sensitive to induced genetic modifications^41,85^.

However, OMVs of all three *X. fastidiosa* strains (Temecula 1, 9a5C, Fb7) have been found to contain as cargo to neighboring and distant cells two of the hydrophobic DSF molecules (XfDSF2 and CVC-DSF), in addition to proteins for virulence and adhesion, such as lipases/esterases and adhesins, among others^32,45^. It has been suggested that the hydrophobic nature of *X. fastidiosa* DSF molecules allows them to embed within the cell membrane, and be subsequently distributed to other cells by release of OMVs^32,45^. Intriguingly, the OMVs secretion itself is also regulated by density-dependent, DSF-based communication^34^. OMVs might thus play a role in initiating and driving filamentous cell growth by binding to the cell membrane of cells in clusters that are either not fully covered by EPS or residing outside the cluster boundaries. In this scenario, the DSF cargo, as well as adhesion-enforcing molecules, could be readily delivered to surrounding bacterial clusters. However, this suggested model, as well as our model of DSF-induced cell morphogenesis, requires further systematic investigation involving alternation of the levels of OMV and diverse DSF candidates^28,71^.

### Implications of DSF-mediated filamentous cell formation for alternative antimicrobial therapies

Despite intensified research and search for therapeutic interventions over the past three decades, to date, no viable commercial therapeutic solution has been found to inhibit the infection in plants or disrupt formed biofilms residing in the plant xylem^62,86^. The common solution to contain the spread of the disease via the insect vector still relies in removing infected plants in a wide geographical radius. Moreover, the development of effective antimicrobial therapies for biofilm-forming bacterial pathogens faces challenges associated with the encapsulation of biofilms in EPS that can act as a diffusion barrier for common antibiotics^87^. This circumstance has led many studies to focus also on alternative strategies to control or disrupt bacterial virulence^40,88,89^.

The prevention of biofilm formation is an alternative approach that more recently emerged based on the findings that cell signaling via quorum-sensing processes is not only associated with the formation of biofilms but is also vital for the processes involved^68,87,90^. For example, knock-out mutants of DSF synthase *rpfF* and sensor kinase *rpfC* have shown, for *X. fastidiosa* Temecula, that blocking the DSF production led to the inability of cells to adhere and form biofilms in the insect vector, whereas the overexpression of DSF resulted in phenotypes in the plants unable to spread^29,30^. Blocking DSF signaling by specifically designed ligands or artificially induced extremely high DSF concentrations would thus disrupt the balance and be effective against disease transmission and spread in infected plants. This approach was successfully tested on *X. fastidiosa* Temecula, where the addition of synthetic signaling molecules inhibited the biological function induced by pathogen ‘confusion’^45,48,89^.

Additionally to the spectrum of DSF molecules identified in *X. fastidiosa*, *Xanthomonas campestris*^91^^,^, and *Stenotrophomonas maltophilia*^48,92^, several other DSF molecules that comprise short saturated and unsaturated fatty acids have been observed in other processes associated with biofilm formation, such as the secretion of EPS, surface adhesion and holdfast, among others^48,69,89,91,93^. This circumstance exemplifies that there might exist many alternative mechanistic targets leading to the disruption of biofilm formation and disease spread by altering the levels of DSF molecules involved in quorum sensing mechanisms. Our identification of cell morphogenesis to filamentous phenotypes playing a key role in the formation of interconnected cell clusters preceding macroscale biofilms provides yet another potential target mechanism for antimicrobial therapies. The blockage of the cell signaling by ligands capturing free DSF molecules or incapacitating the associated sensory kinase ligands would result in the inability for *X. fastidiosa* to form large biofilms. However, for all these exemplified scenarios, further investigations of the mechanisms underlying the different DSF expression and sensory systems are necessary.

In conclusion, while morphological plasticity and filamentous phenotypes have been reported for a variety of other bacteria, their morphogenesis was predominantly associated with adaptive responses to environmental changes and diverse forms of stress including nutrition deprivation. In contrast to this common belief, our results shed light on how the formation of filamentous cells plays instead a vital role in *X. fastidiosa* biofilm formation per se. In turn, considering that filamentous cell formation has been observed for diverse bacteria and that *X. fastidiosa* shares major genetic traits with other human and plant bacteria, DSF-mediated quorum sensing might represent a conserved regulatory mechanism existing in other biofilm-forming bacterial pathogens. In this context, our findings open pathways for the identification of alternative targets in biofilm-forming pathogens and the design of quorum sensing inhibitors to eventually inhibit biofilm formation.

## Materials and methods

### Bacteria strains

*X. fastidiosa* pauca 11399 strain^41,85^ expressing soluble GFP was used in this study^17^. To prepare the pre-inoculum^17,41^, the bacterial cells grown on solid Periwinkle Wilt broth (PW) plates^94^ were harvested after 7 days and resuspended in PBS buffer. The bacteria cell culture was adjusted to an optical density of 0.5 OD_600 nm_ with fresh PW broth and grown at 28 °C for 7 days while shaking at 150 rpm or estimated 0.15 × *g*.

### Bacterial growth

Bacterial inoculum with a concentration of 1 × 10^7^ CFU/mL from the pre-inoculum was used for the experiments as initial concentration for bacterial growth studies in PW broth media^94^. The substrates with Au arrays were stored during bacterial growth inside sterile Petri dishes. 600 µL of bacteria inoculum together with 2400 µL of fresh PW growth media were added to the Petri dishes containing the substrates, which were subsequently sealed with parafilm. The samples were placed in a bacterial incubator (410/3NDR, Nova Ética, Brazil) during bacterial growth at 28 °C without shaking and culture media replacement (unless specified in the respective manuscript text) for different growth times (specified in the respective manuscript text). For the experiments involving the addition of supernatant extract or PW replenishment, the equal volume of the PW growth media is removed from the Petri dish before the addition of either the supernatant extract (2% v/v) or fresh PW (20% v/v) after 4 or 8 h of growth for the experiments with a growth time of 8 or 18 h, respectively.

After certain growth times (6, 8, and 18 h) the PW broth media was removed gently and the samples were then washed twice with DI water to remove remaining chemical compounds of the culture media as well as non-attached bacteria. In a final step, the samples were dried gently with a nitrogen flow and temporarily stored at 4 °C before fluorescence measurement.

### Supernatant extract

*X. fastidiosa* wild-type strain 11399 grown on solid PW^94^ were harvested and resuspended in 1 mL PBS buffer to an OD_600_ of 1.5, inoculated into 9 mL PW broth without bovine serum albumin (BSA), and grown at 28 °C for 10 days while shaking at 150 rpm or estimated 0.15 × *g*. The bacterial suspensions were then collected, filtered through a 0.22 µm filter^32^, and stored at −80 °C until the experiments.

### Fabrication of Au microarray patterns

The Au micro-patterns are fabricated by photolithography on cleaned borosilicate SiO_2_ substrates. Mask-free direct laser writing (DWL), equipped with a 380 nm solid-state laser (Heidelberg Instruments µpg10; Power: 6 mW), was used on spin-coated AZ5214E photo resist (5000 rpm—or estimated 340 × *g*—for 50 s, providing a resist layer with ~1 µm thickness). After lithography, the patterns were developed using AZ351B (4:1) developer solutions for 15 s. Afterwards, 20 nm-thick Au coating was deposited by electron beam physical vapor deposition (ULS600, Oerlikon Balzers, Liechtenstein) at 5 × 10^−7^ Torr. Finally, photoresist lift-off was carried by sonication with acetone for 2 min and rinsing with isopropanol and deionized (DI) water. The substrates were sterilized with oxygen plasma (SE80, Barrel Asher Plasma Technology, USA) for 10 min (100 mT, 50 sccm, 200 W) right before the bacterial adhesion experiments.

### Wide-field epifluorescence microscopy

Dried bacteria samples of different growth times (6, 8, and 18 h) were measured using an epifluorescence microscope (Nikon TE2000U, USA) with a peltier-cooled back-illuminated EMCCD camera (IXON3, 1024 × 1024 pixels, Andor, Ireland) and a ×60 water-immersion objective (CFI APO, NA 1.2, Nikon USA). GFP excitation and bacterial bright-field imaging were achieved by a 150 W Mercury-lamp with filter sets (AHF, Tübingen, Germany) for GFP (488 nm) and neutral density (ND8) filters, respectively. For each bacterial sample, a bright-field and a fluorescence image were taken sequentially. The images were merged and analyzed using Fiji/ImageJ software^95^.

### Confocal laser scanning microscopy

For the in-vitro CLSM studies, the samples were placed inside a Teflon dish liquid cell (10 mm diameter and 5 mm in height), covered with a sterilized borosilicate cover glass. For each measurement, 400 µL of four times diluted *X. fastidiosa* 11399 inoculums was injected inside the liquid cell and incubated at 28 °C for 14 h. CLSM measurements were performed using a Zeiss LSM780-NLO Confocal microscope (Carl Zeiss AG, Germany) with a ×40 water-immersion objective (Plan-Apochromat, NA. 1.0, Zeiss) for in vitro studies, and a ×20 long-distance objective (Plan-Nanofluar, NA 0.5, Zeiss) for dried samples. The reflection of Au patterns and the fluorescence of GFP bacteria cells were simultaneously measured in two different channels. The GFP excitation was performed with a 488 nm laser line and the position of Au arrays was identified by the reflected laser. Imaging was performed with pinholes set to 1 airy unit for each channel and with a 512 × 512 px image resolution.

### Image analysis

The images were merged and analyzed using Fiji/Imagej software^95^. The measured fluorescence intensity, area of bacterial adhesion, as well as integrated fluorescence density were extracted from raw fluorescence, reflective or bright-field images using in-built scripts for threshold and area measurement of the Fiji/ImageJ software package. Background subtraction was performed on each individual fluorescence image.

### Reporting summary

Further information on research design is available in the Nature Research Reporting Summary linked to this article.

## Supplementary information

Supplementary information

Reporting Summary

## Data Availability

The authors declare that all the data supporting the findings of this study are available within the article and its Supplementary Information. Raw data are also available from the corresponding authors upon request.
