# Supplementary material for: Controlled spatial organization of bacterial growth reveals key role of cell filamentation preceding Xylella fastidiosa biofilm formation
Source: NPJ Biofilms Microbiomes. 2021 Dec 7;7:86. doi: 10.1038/s41522-021-00258-9 (PMC8651647; doi:10.1038/s41522-021-00258-9)
Supplement: Supplementary file 1 — Supplementary information [file 41522_2021_258_MOESM1_ESM.pdf]

Supplementary Material for:

Controlled spatial organization of bacterial growth reveals key role of cell filamentation preceding *Xylella fastidiosa* biofilm formation

Silambarasan Anbumani[1], Aldeliane M. da Silva,[1] Isis G. B. Carvalho[2], Eduarda Regina Fischer[2], Mariana de Souza e Silva[2], Antonio Augusto G. von Zuben[1], Hernandes F. Carvalho[3], Alessandra A. de Souza[2], Richard Janissen[4,] and Monica A. Cotta[1,*]*

[1]Institute of Physics "Gleb Wataghin", University of Campinas-13083-859 Campinas, São Paulo, Brazil.
[2]Citrus Center APTA "Sylvio Moreira" Agronomic Institute of Campinas 13490-970 Cordeirópolis, São Paulo, Brazil.
[3]Department of Structural and Functional Biology, Institute of Biology, University of Campinas, 13083-862, Campinas, São Paulo, Brazil.
[4]Kavli Institute of Nanoscience, Delft University of Technology, 2629 HZ Delft, The Netherlands.

*Corresponding authors: Richard Janissen, Email: r.janissen@tudelft.nl; Monica A. Cotta, Email: monica@ifi.unicamp.br

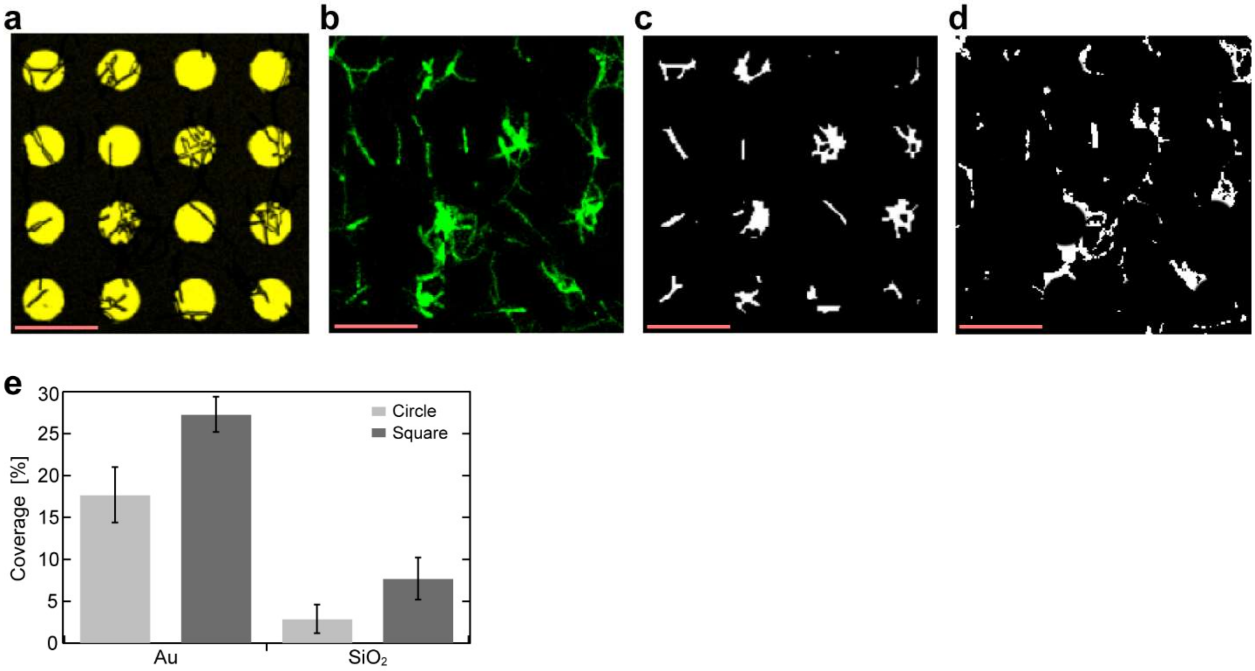

Supplementary Figure S1: (**a**) Bright-field image of circular shaped Au patterns with adhered *X. fastidiosa*, and (**b**) corresponding fluorescence image after 18 h of growth. (**c**) Binary image of adhered *X. fastidiosa* on Au disks by extracting the areas of the Au disks shown in (a). (**d**) Binary image of adhered *X. fastidiosa* on SiO_2 surface by subtracting from the fluorescence image (B) the areas of the Au disks shown in (a). Scale bar depicts 20 µm. (**e**) *X. fastidiosa* coverage area (in % of total Au or SiO_2 area; mean±s.d.) on circular- and square-shaped Au surfaces, as well as on the SiO_2 surfaces, measured from data shown in Figure 1d. **Related to Figure 1**.

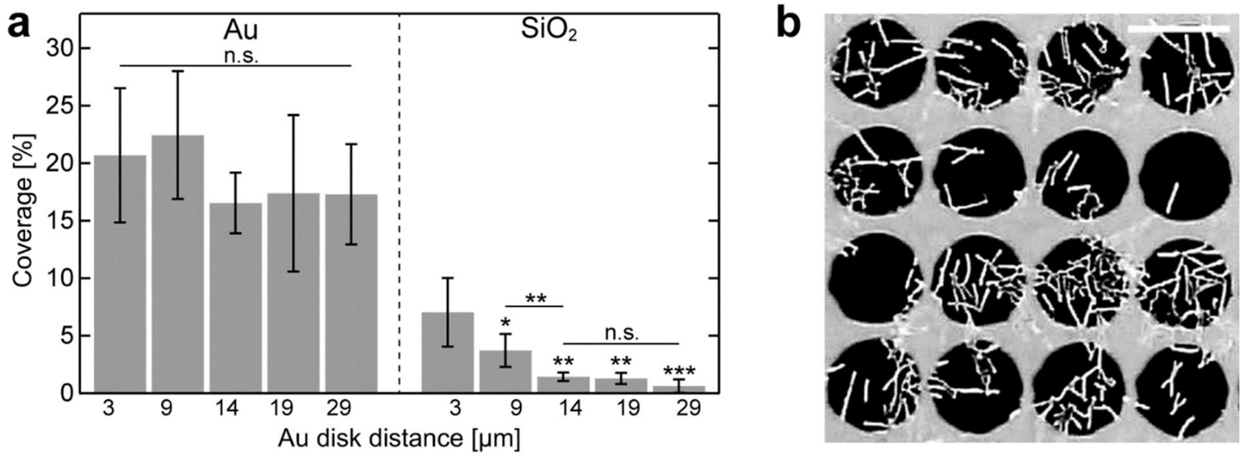

Supplementary Figure S2: (**a**) *X. fastidiosa* coverage area (in % of total Au or SiO_2 area; mean±s.d.) on Au and SiO_2 surfaces, measured for different Au disk distances; related to Figure 2c. All statistical analyses consisted of unpaired, two-tailed t-tests (***$p < 0.001$; **$p < 0.01$; n.s. = non-significant). (**b**) Bright-field image of adhered *X. fastidiosa* on circular-shaped Au disks with 11 µm in diameter and 3 µm spacing between the disks after 8 hours of growth; scale bar depicts 15 µm. **Related to Figure 2**.

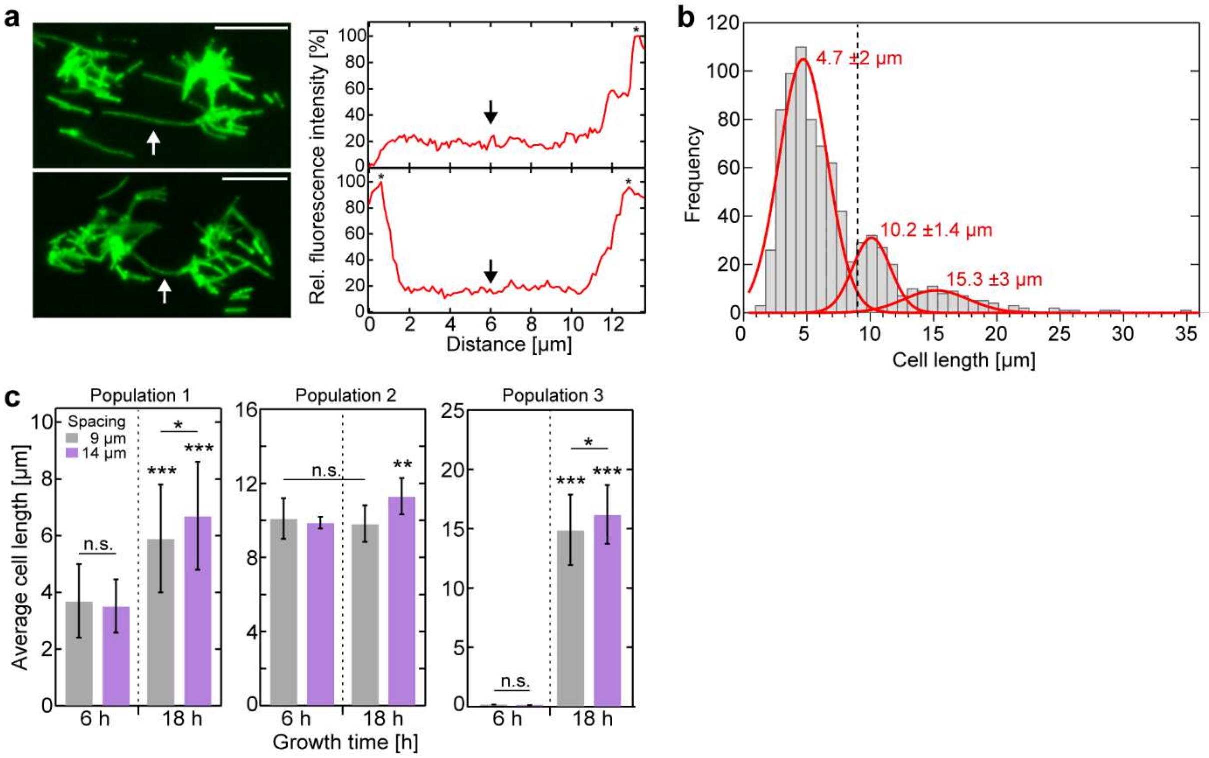

Supplementary Figure S3: (**a**) Representative fluorescence images (left) of filamentous cells (indicated by white arrows) emanating from bacterial cell clusters, interconnecting adjacent cell clusters. Corresponding background-corrected fluorescence intensity profiles (right) along the filamentous cells; scale bar depicts 10 µm. Asterisks on the intensity profiles represent the *X. fastidiosa* cluster boundary. Based on the results shown in (a) and (b), cells with lengths >9 µm and no significant GFP fluorescence signal variation along their cell body are considered as filamentous cells for analysis. (**b**) *X. fastidiosa* cell length distribution (N = 833) pooled from experiments including both 6 and 18 h growth times, and 9 and 14 µm spacing between Au disks with 11 µm in diameter. Gaussian mixture model results in three distinct Gaussian fits (red lines), indicating three cell length populations. Dotted line indicates Gaussian peak -σ of the second population, determining a cell length threshold of 9 µm between typical cell lengths and cell length of filamentous cells. (**c**) Comparison of N = 228 average cell lengths (mean±s.d.), separated by the three different cell length populations identified in Figure 3a (***p < 0.001), resulting from two-tailed, unpaired t-tests. **Related to Figure 3**.

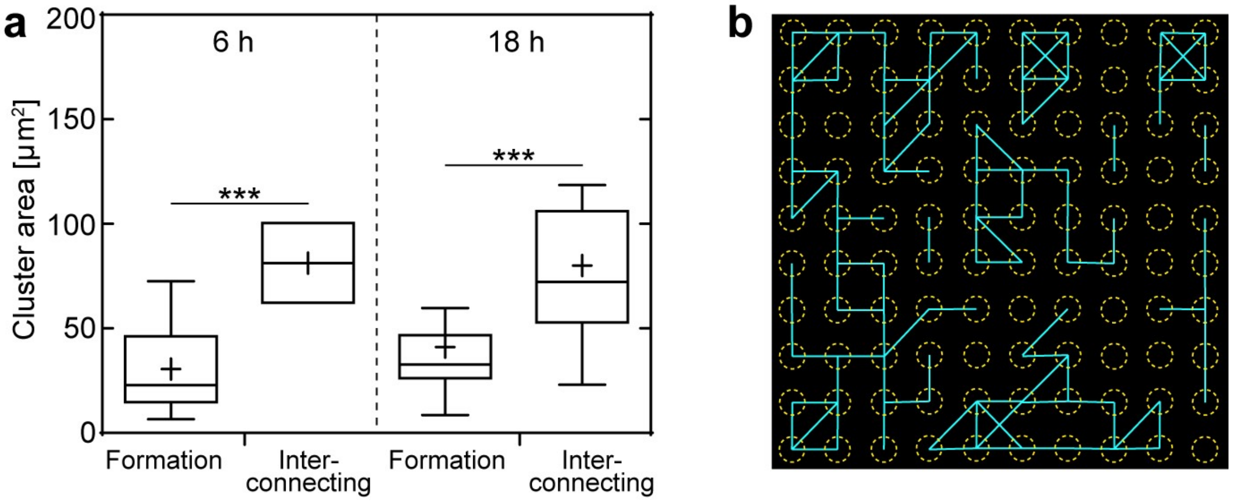

Supplementary Figure S4: (**a**) Area distribution for bacterial cell clusters that solely form filamentous cells or form filamentous cell that interconnect neighboring clusters, for 6 h and 18 h growth time (Formation: N = 33 (6 h), N = 40 (18 h); Interconnecting: N = 6 (6 h), N = 17 (18 h)). Statistical analyses consisted of unpaired, two-tailed t-tests (***$p < 0.001$). Legend for box and whisker plot: the center line denotes the median value (50th percentile), the cross the mean average, the bounds of box contain the 25th to 75th percentiles, the whiskers mark the 1.5 interquartile range. (**b**) Schematics of detected interconnections (cyan) of neighboring cell clusters (adhered to Au disks; yellow) by filamentous cells fluorescence image shown in Figure 4d. **Related to Figure 4**.

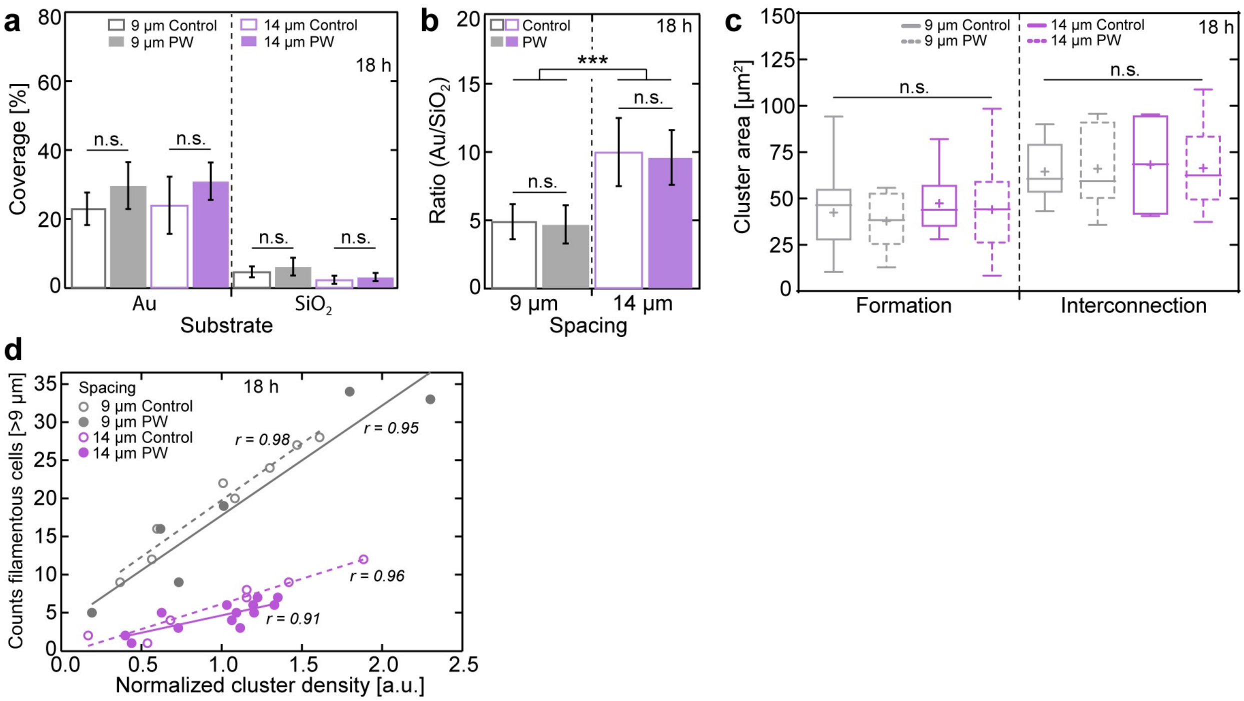

Supplementary Figure S5: (**a**) *X. fastidiosa* coverage area (mean±s.d.) on Au and SiO_2 surfaces after 18 h of growth, measured for 9 and 14 μm Au disk distances, and in absence or addition of 20% (v/v) Periwinkle Wilt broth after 9 h (PW). (**b**) Ratio (mean±s.d.) of Au to SiO_2 bacterial coverage extracted from (a). (**c**) Bacterial cluster area-dependent *formation* of filamentous cells and filamentous cells that *interconnect* adjacent bacterial clusters for 9 and 14 μm spacing samples grown for 18 h, in absence or addition of 20% (v/v) Periwinkle Wilt broth after 9 h (PW). Legend for box and whisker plot: the center line denotes the median value (50th percentile), the cross the mean average, the bounds of box contain the 25th to 75th percentiles, the whiskers mark the 1.5 interquartile range. (**d**) Number of filamentous cells in dependency of cluster density after 18 h growth, measured for 9 and 14 μm Au disk distances, and in absence or addition of 20% (v/v) Periwinkle Wilt broth after 9 h (PW). Guide lines represent linear fits resulting from Pearson correlation fits; the coefficients r are denoted in the legends for each fit. Statistical analyses in (a), (b), and (c) consisted of unpaired, two-tailed t-tests (***$p < 0.001$; n.s. = non-significant). **Related to Figure 5**.

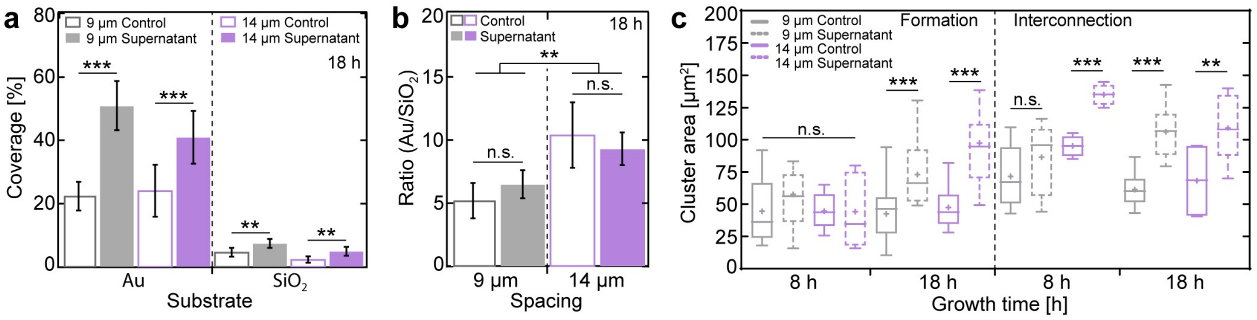

Supplementary Figure S6: (**a**) *X. fastidiosa* coverage area (in % of total Au or SiO_2 area; mean±s.d.) on Au and SiO_2 surfaces after 18 h of growth, measured for 9 and 14 µm Au disk distances, and in absence or addition of 2% (v/v) of supernatant after 9 h. (**b**) Ratio (mean±s.d.) of Au to SiO_2 bacterial coverage extracted from (a). (**c**) Bacterial cluster area-dependent *formation* of filamentous cells and filamentous cells that *interconnect* adjacent bacterial clusters for 9 and 14 µm spacing samples and grown for 8 and 18 h, and in absence or addition of 2% (v/v) of supernatant after 9 h. Legend for box and whisker plot: the center line denotes the median value (50th percentile), the cross the mean average, the bounds of box contain the 25th to 75th percentiles, the whiskers mark the 1.5 interquartile range. All statistical analyses consisted of unpaired, two-tailed t-tests (***$p < 0.001$; **$p < 0.01$; n.s. = non-significant). **Related to Figure 5**.
